# m^6^A Modification Involves in Enriched Environment-Induced Neurogenesis and Cognition Enhancement

**DOI:** 10.3389/fcell.2022.903179

**Published:** 2022-06-02

**Authors:** Wenzheng Qu, Qian Li, Mengxuan Wang, Xingsen Zhao, Jiangdong Wu, Diwen Liu, Shenghui Hong, Ying Yang, Qiang Shu, Xuekun Li

**Affiliations:** ^1^ The Children’s Hospital, National Clinical Research Center for Child Health, School of Medicine, Zhejiang University, Hangzhou, China; ^2^ The Institute of Translational Medicine, School of Medicine, Zhejiang University, Hangzhou, China; ^3^ Laboratory Animal Center, Zhejiang University, Hangzhou, China; ^4^ CAS Key Laboratory of Genomic and Precision Medicine, Collaborative Innovation Center of Genetics and Development, College of Future Technology, Beijing Institute of Genomics, Chinese Academy of Sciences, Beijing, China; ^5^ University of Chinese Academy of Sciences, Beijing, China; ^6^ Zhejiang University Cancer Center, Zhejiang University, Hangzhou, China

**Keywords:** N6-methyladenosine RNA methylation, enriched environment, fat mass and obesity-associated gene, neuronal development, neural stem/progenitor cell, neurogenesis, cognition

## Abstract

Although previous studies have shown that an enriched environment (EE) promotes neurogenesis and alters DNA and histone modifications, it remains largely unknown whether an EE affects epitranscriptome in the context of neuronal development. Here, we showed that EE exposure enhanced the pool of adult neural stem/progenitor cells (aNSPCs) and promoted neuronal differentiation of aNSPCs. EE exposure also improved cognitive capabilities and altered the expression of genes relating to neuronal development, neurogenesis, and memory. *N* ^6^-Methyladenosine (m^6^A) immunoprecipitation combined with deep sequencing (MeRIP-seq) data analysis revealed that EE exposure increased the global level of m^6^A and led to differential m^6^A mRNA modification. Differential m^6^A modification-associated genes are involved in neuronal development, neurogenesis, and so on. Notably, EE exposure decreased the protein level of m^6^A eraser Fto, but did not affect the protein level of m^6^A writers METTL3 and METTL14. Taken together, our results suggest that enriched environment exposure induces differential m^6^A mRNA modification and adds a novel layer to the interaction between the environment and epigenetics in the context of postnatal neuronal development.

## Introduction

During the postnatal neuronal development, mammalian neurogenesis driven by neural stem/progenitor cells (aNSPCs) is modulated by diverse factors including environment, genetics, and epigenetics (Li and Jin, 2010; Ma et al., 2010; Sun et al., 2014; Avgustinova and Benitah, 2016; Hsieh and Zhao, 2016; Yao et al., 2016; Atlasi and Stunnenberg, 2017). As the most abundant RNA modification in mRNAs of eukaryotic cells, *N* ^*6*^-methyladenosine (m^6^A) modification is deposited by methyltransferase-like 3 (METTL3)-methyltransferase-like 14 (METTL14) complex and can be erased by fat-mass and obesity-associated protein (Fto) and α-ketoglutarate-dependent dioxygenase alkB homolog 5 (Alkbh5) in mRNAs of eukaryotic cells. Previous studies have shown that m^6^A modification involves a variety of biological processes including cell fate determination, proliferation and differentiation of stem cells, circadian, homeostasis, DNA damage response, and adipogenesis (Fustin et al., 2013; Batista et al., 2014; Wang et al., 2014; Zhao et al., 2014; Li et al., 2017a; Zhang et al., 2017).

m^6^A displays dynamic features during the embryonic and postnatal neuronal development, and neurodegeneration in mammals (Meyer et al., 2012; Shafik et al., 2021; Zhao et al., 2021). The modulation of *Mettl14* or *Mettl3* decreases the level of m^6^A and consequently regulates embryonic and adult neurogenesis, cerebellar development, and stress response (Wang et al., 2018a; Wang et al., 2018b; Engel et al., 2018; Li et al., 2018; Ma et al., 2018; Shi et al., 2018; Chen et al., 2019; Cao et al., 2020; Gao et al., 2020; Livneh et al., 2020). In addition, constitutive and specific modulation of *Fto* in differential lineages of cells affects neuronal activity, depression, fear, and spatial memory of mice (Hess et al., 2013; Sevgi et al., 2015; Nainar et al., 2016; Widagdo et al., 2016; Li et al., 2017b; Cao et al., 2020; Gao et al., 2020; Liu et al., 2021). In line with its effects on transcriptome, differential mechanisms for m^6^A playing function have been revealed, such as interacting with histone methyltransferase EZH2, increasing adenosine, and regulating BDNF signaling pathway (Li et al., 2017b; Chen et al., 2019; Gao et al., 2020).

The environment is critical for postnatal neuronal development, and epigenetics, such as DNA and histone modifications, mediate gene-environment interaction. An enriched environment (EE), in which animals are usually housed in a transparent larger cage equipped with toys, tunnels, and running wheels, which involves sensorimotor and social stimulation, shows remarkable influences on neuronal plasticity, neurogenesis, and hippocampus-dependent learning and memory (Kempermann, 2019; Zocher et al., 2020; Gronska-Peski et al., 2021; Zocher et al., 2021; Lupori et al., 2022). EE exposure counteracts age-related DNA methylation (Zocher et al., 2021), DNA hydroxymethylation (Irier et al., 2014), and regulates gene expression including fibroblast growth factor receptor (FGFR) (Rampon et al., 2000; Kempermann, 2019; Gronska-Peski et al., 2021). However, it remains largely unknown regarding the effects of EE exposure on m^6^A mRNA modification in the context of neuronal development.

In the present study, we found that enriched environment treatment enhanced the pool and promoted neuronal differentiation of adult neural stem/progenitor cells (aNSPCs). Behavioral tests showed that EE exposure also enhanced the cognitive capabilities of mice. RNA-seq data analysis showed that EE exposure altered the expression of genes relating to neuronal development, neurogenesis, and memory. MeRIP-seq data analysis revealed that EE exposure increased the global level of m^6^A modification. Differential m^6^A modification-associated genes are involved in neuronal development, neurogenesis, and so on. Notably, EE exposure decreased Fto protein level but did not affect METTL3 and METTL14 protein levels. Taken together, our results revealed that an enriched environment induces differential m^6^A mRNA modification and regulates the proliferation and differentiation of aNSPCs and cognition of mice during postnatal neuronal development.

## Results

### Enriched Environment Promotes Mouse Hippocampal Neurogenesis and Cognition

To examine the effects of enriched environment exposure on postnatal hippocampal neurogenesis, wild-type (WT, C57BL6) mouse pups (postnatal day 21) were weaned and housed in standard (SH, 4–5 animals per cage) and enriched environment (EE, 8–9 animals per cage), respectively (Supplementary Figure S1A). Five weeks later, neurogenesis assay, MeRIP-seq/RNA-seq, and behavioral tests were performed (Supplementary Figure S1B).

**FIGURE 1:**
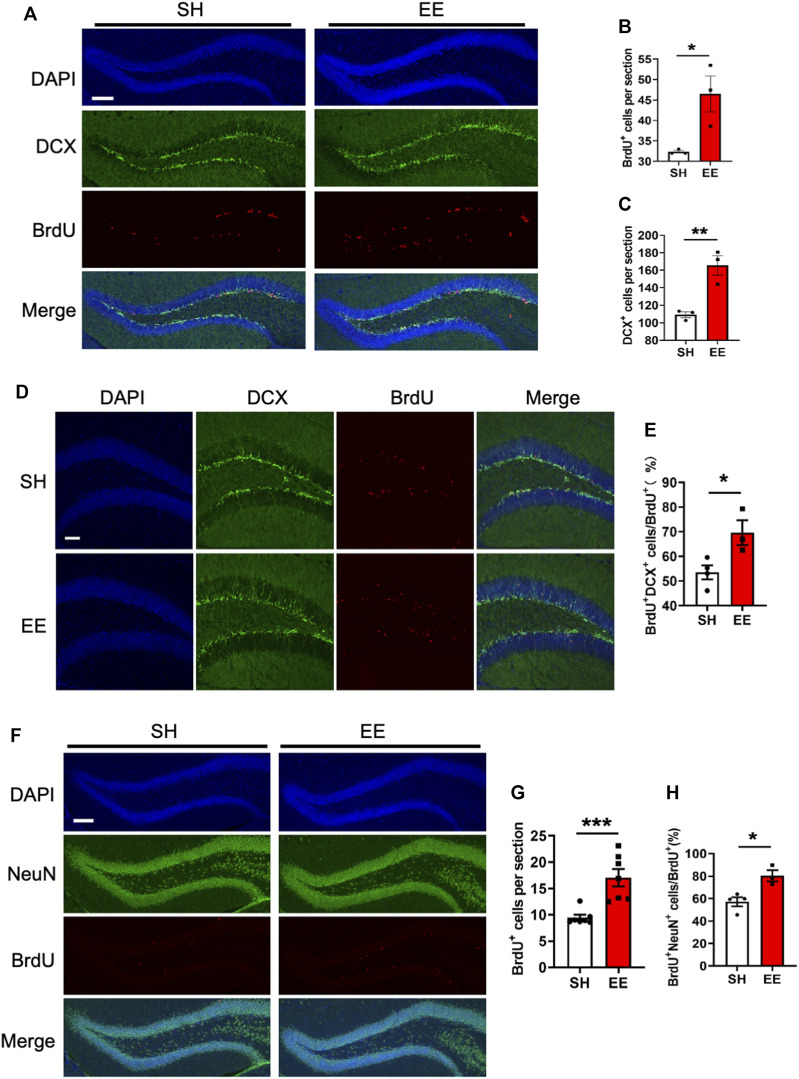
Enriched environment exposure promotes postnatal hippocampal neurogenesis. **(A)** Representative images of BrdU and DCX immunostaining with the brain sections of SH and EE mice, respectively. Adult mice were administrated with BrdU six times with an interval of 4 h and sacrificed 4 h post the final BrdU administration. BrdU, 5-bromo-2′-dexoyuridine. Scale bar, 50 μm. **(B**,**C)** Quantification results showed that EE exposure significantly increased the numbers of BrdU^+^ **(B)** and DCX^+^ **(C)** cells compared to SH mice, respectively. EE/SH, *n* = 3 mice. Values represent mean ± SEM; *p < 0.05; **p < 0.01; ***p < 0.001; unpaired Students t-test. DCX, doublecortin. **(D)** Representative images of BrdU-DCX immunostaining with the brain sections of SH and EE mice, respectively. Mice were administrated with BrdU six times, two times per day for three consecutive days with an interval of 12 h, and sacrificed 1-week post the final BrdU administration. Scale bar, 50 μm. **(E)** Quantification results showed that EE exposure significantly increased the percentage of BrdU^+^DCX^+^ cells compared to SH mice. SH, *n* = 4 mice; EE, *n* = 3 mice. Values represent mean ± SEM; *p < 0.05; **p < 0.01; ***p < 0.001; unpaired Students t-test. **(F)** Representative images of BrdU-NeuN immunostaining with the brain sections of SH and EE mice, respectively. Mice were sacrificed 4-week post the final BrdU administration. Scale bar, 50 μm. NeuN, neuronal nuclei. **(G**,**H)** Quantification results showed that EE exposure significantly increased the number of BrdU^+^ cells **(G)** and the percentage of BrdU^+^NeuN^+^ cells compared to SH mice, respectively. SH, *n* = 4 mice EE, *n* = 3 mice. Values represent mean ± SEM; *p < 0.05; **p < 0.01; ***p < 0.001; unpaired Students t-test.

To analyze the effects of EE exposure on the proliferation of adult neural stem/progenitor cells (aNSPCs), animals were injected with BrdU and sacrificed 1-day post the final BrdU administration (Supplementary Figure S1C). Immunofluorescence staining and quantification results showed that EE exposure significantly increased the number of BrdU positive (BrdU^+^) cells (proliferation assay) compared to that of the SH group (Figures 1A,B). In addition, the number of neural progenitor cells marker Doublecortin (DCX) positive (DCX^+^) cells also was significantly increased (Figures 1A,C). To analyze the effects of EE exposure on the differentiation of aNSPCs, animals were sacrificed at the time-point of 1-week post the final BrdU administration (Supplementary Figure S1C). Immunofluorescence staining and quantification results showed that EE exposure led to a remarkable increase in the percentage of BrdU^+^DCX^+^/BrdU^+^ (Figures 1D,E). Furthermore, we observed that the number of BrdU^+^ cells and the percentage of newborn mature neurons (BrdU^+^NeuN^+^/BrdU^+^) both significantly increased in the brain of EE-exposed mice compared to those of SH mice 1 month post the final BrdU administration (Figures 1F–H). Taken together, these results suggest that EE exposure enhances the pool of aNSPCs and promotes adult neurogenesis.

### Enriched Environment Exposure Enhances the Cognitive Function of Mice

**FIGURE 2:**
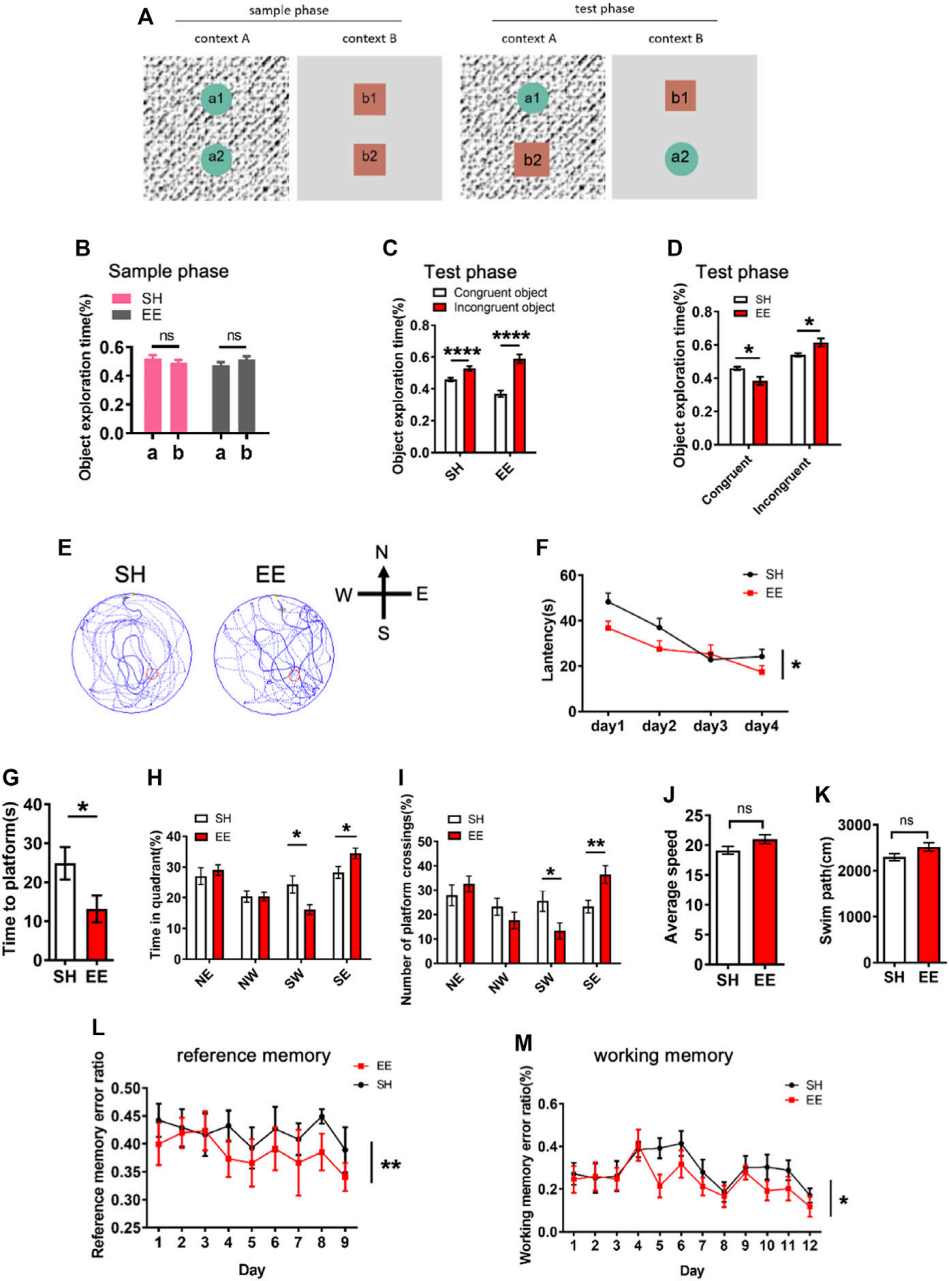
Enriched environment exposure enhances the learning and memory of mice. **(A)** Schematic illustration of pattern separation memory test. In the sample phase, mice were sequentially exposed to two similar but distinct contexts one and two containing pairs of identical objects. In the test phase, mice were placed in either context one or two containing one congruent object and one incongruent object. **(B)** Pattern separation memory test showed that SH and EE mice did now show a difference in the proportion of object exploration time during the sample phase. EE mice, *n* = 14; SH mice, *n* = 12. Values represent mean ± SEM; ns, not significant; *p < 0.05; **p < 0.01; ***p < 0.001; unpaired Students t-test. **(C**,**D)** Pattern separation memory test showed that SH and EE mice spent a greater proportion of time exploring the incongruent object **(C)**, but EE mice exhibit a higher proportion of time for an incongruent object **(D)** compared to SH mice during the test phase. EE mice, *n* = 14; SH mice, *n* = 12. Values represent mean ± SEM; *p < 0.05; **p < 0.01; ***p < 0.001; ****p < 0.0001; unpaired Students t-test. **(E)** Representative images of the swimming path of SH and EE mice during the testing period in the Morris Water Maze test. **(F)** EE mice displayed shorter escape latency compared to SH mice during the training period in the Morris Water Maze test. SH, *n* = 7 mice; EE, *n* = 8 mice. Values represent mean ± SEM; *p < 0.05; **p < 0.01; ***p < 0.001; ****p < 0.0001; unpaired Students t-test. **(G**–**K)** Probe trial test results showed that EE mice displayed a shorter time to reach the platform **(G)**, longer time in the target quadrant field **(H)**, and more numbers of platform crossings **(I)**, while SH and EE mice exhibited similar swimming speed **(J)** and length **(K)**. SH, *n* = 7 mice; EE, *n* = 8 mice. Values represent mean ± SEM; *p < 0.05; **p < 0.01; ***p < 0.001; ****p < 0.0001; unpaired Students t-test. **(L**,**M)** Eight-arm maze test results showed that EE mice displayed lower error ratios of reference **(L)** and working **(M)** memory. SH, *n* = 7 mice; EE, *n* = 8 mice. Values represent mean ± SEM; *p < 0.05; **p < 0.01; ***p < 0.001; ****p < 0.0001; unpaired Students t-test.

We next performed behavioral tests to examine whether EE exposure affected the cognition of mice. For the pattern separation test, SH and EE-exposed mice were first tested in an object-context discrimination task (Figure 2A). We observed that SH and EE-treated mice showed similar proportions of time exploring the objects during a sample phase (Figure 2B). During the test phase, SH and EE-exposed mice spent more time exploring the incongruent object (Figure 2C). However, EE-treated mice showed less time exploring the congruent object and more time exploring the incongruent object compared to SH mice during the test phase (Figure 2D).

Furthermore, we performed a morris water maze (MWM) test and observed that EE-exposed mice displayed a shorter latency during the training period (Figures 2E,F). The probe trial test showed that EE-treated mice showed shorter latency, increased time in the target quadrant, and numbers of crossing the platform (Figures 2G–I), though SH and EE-treated mice exhibited similar swimming paths length and speed (Figures 2J,K). In addition, the eight-arm maze test showed that EE-exposed mice showed reduced working and reference memory error ratios compared to those of SH mice (Figures 2L,M). Collectively, these results suggest that EE exposure enhances the cognitive function of mice during postnatal neuronal development.

### Enriched Environment Treatment Alters the Neuronal Transcriptome

**FIGURE 3:**
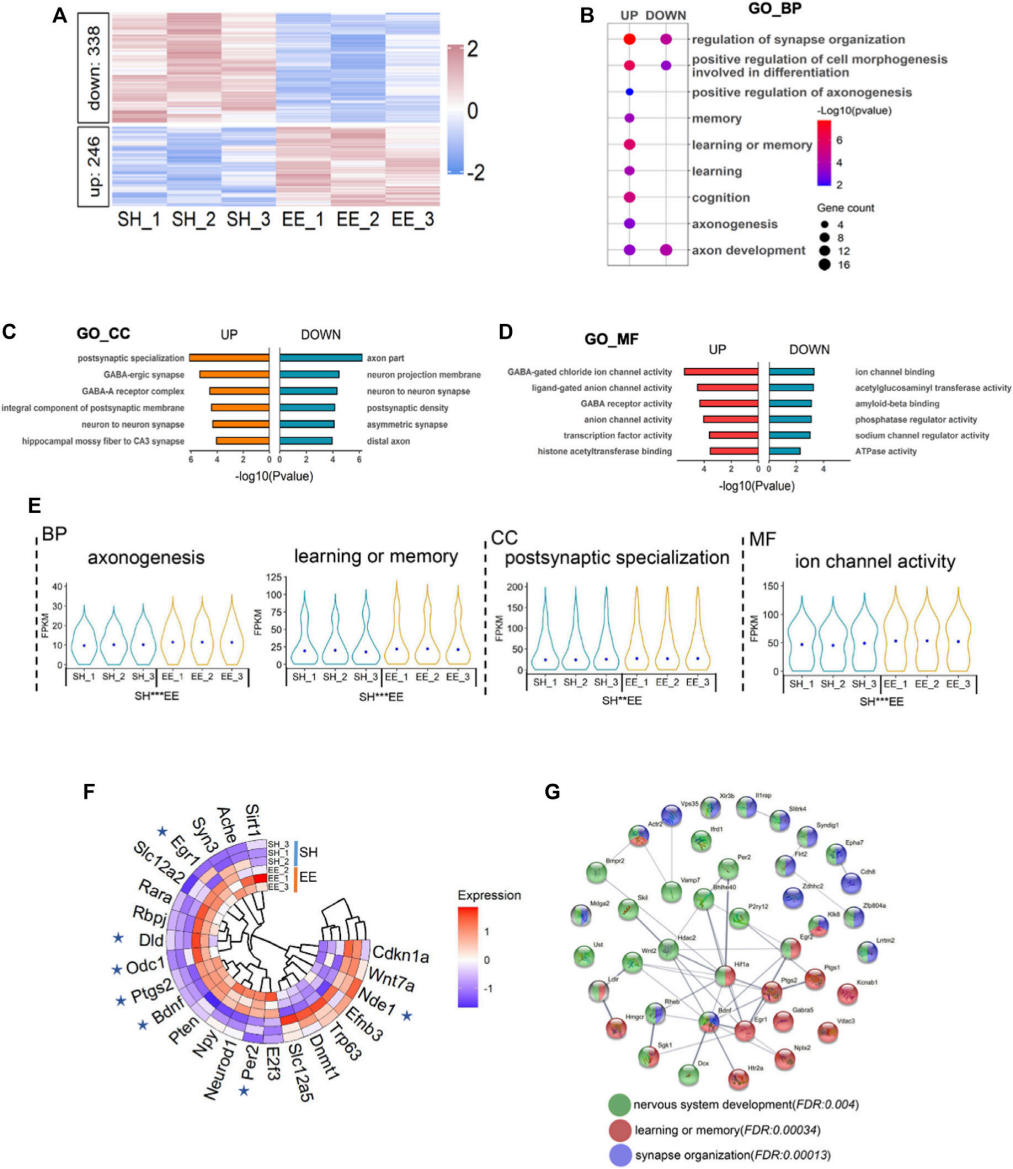
Enriched environment exposure leads to the altered transcriptome. **(A)** Heatmap illustrating the altered transcriptome in EE mice compared to SH mice. Red color, up-regulated genes; blue color, down-regulated genes. Three biological repeating brain samples of SH and EE mice were adopted for sequencing. **(B)** GO analysis of the up- and down-regulated genes revealed enrichment for biological process terms related to synapse organization, axon development, learning, and memory. **(C**,**D)** GO cell component (GO_CC) **(C)** and GO molecular function (GO_MF) enrichment analysis showed that differential expressed genes (DEGs) involve in synapse activity, especially GABAergic synapse. **(E)** Violin plots showed that up-regulated genes in the EE group significantly enriched in the indicated GO terms compared to the SH group. *p < 0.05; **p < 0.01; ***p < 0.001; unpaired Students t-test. **(F)** Twenty-three genes in the MANGO database were revealed by our RNA-seq. Seven genes, six up- and one down-regulated, exhibited altered expression. **(G)** The interaction analysis showed up-regulated genes related to neuronal development (green), learning and memory (red), and synapse organization (purple).

To uncover the molecular mechanism underlying EE exposure regulation of postnatal neurogenesis, we performed RNA-seq with RNA extracted from the hippocampi tissue of SH and EE mice, respectively. Gene set enrichment analysis (GSEA) of RNA-seq data showed that all expressed genes were enriched in gene transcription and long-term memory (Supplementary Figures S2A,B). The expression of 584 genes significantly alternated, 246 up- and 338 down-regulated, in the EE group compared to the SH group (Figure 3A and Supplementary Table S1). Gene ontology (GO) analysis revealed that up-regulated genes significantly enriched in terms relating to neuronal development, learning, cognition, and axon development, et al. (Figure 3B). Cell component and molecular function enrichment analysis indicated that up- and down-regulated genes were involved in synapse development and neuronal activity (Figures 3C,D). Notably, compared to SH mice, up-regulated genes induced by EE exposure were significantly enriched in terms including axonogenesis, learning or memory, postsynaptic specialization, and ion channel activity (Figure 3E).

We further performed an integrated analysis of the differentially expressed genes (DEGs) revealed by our RNA-seq with the genes in the MANGO database, which annotated 263 genes promoting the hippocampal neurogenesis (Overall et al., 2012). 23 genes revealed by our RNA-seq were identified in this database, and 7 of 23 genes showed the altered expression (Figure 3F and Supplementary Table S2). Six genes including *Egr1*, *Dld*, *Odc1*, *Ptgs2*, *Bdnf*, *Per2* were remarkably up-regulated, and one gene, *Nde1*, was significantly down-regulated in the EE group compared with the SH group (Figure 3F). In addition, protein interaction analysis showed that up-regulated genes coding proteins were enriched in terms of neuronal system development, learning and memory, synapse organization, and neurogenesis (Figure 3G). Together, these results suggest that EE exposure promotes the expression of genes associated with neurogenesis.

### Enriched Environment Exposure Leads to the Alteration of m^6^A Modification

**FIGURE 4:**
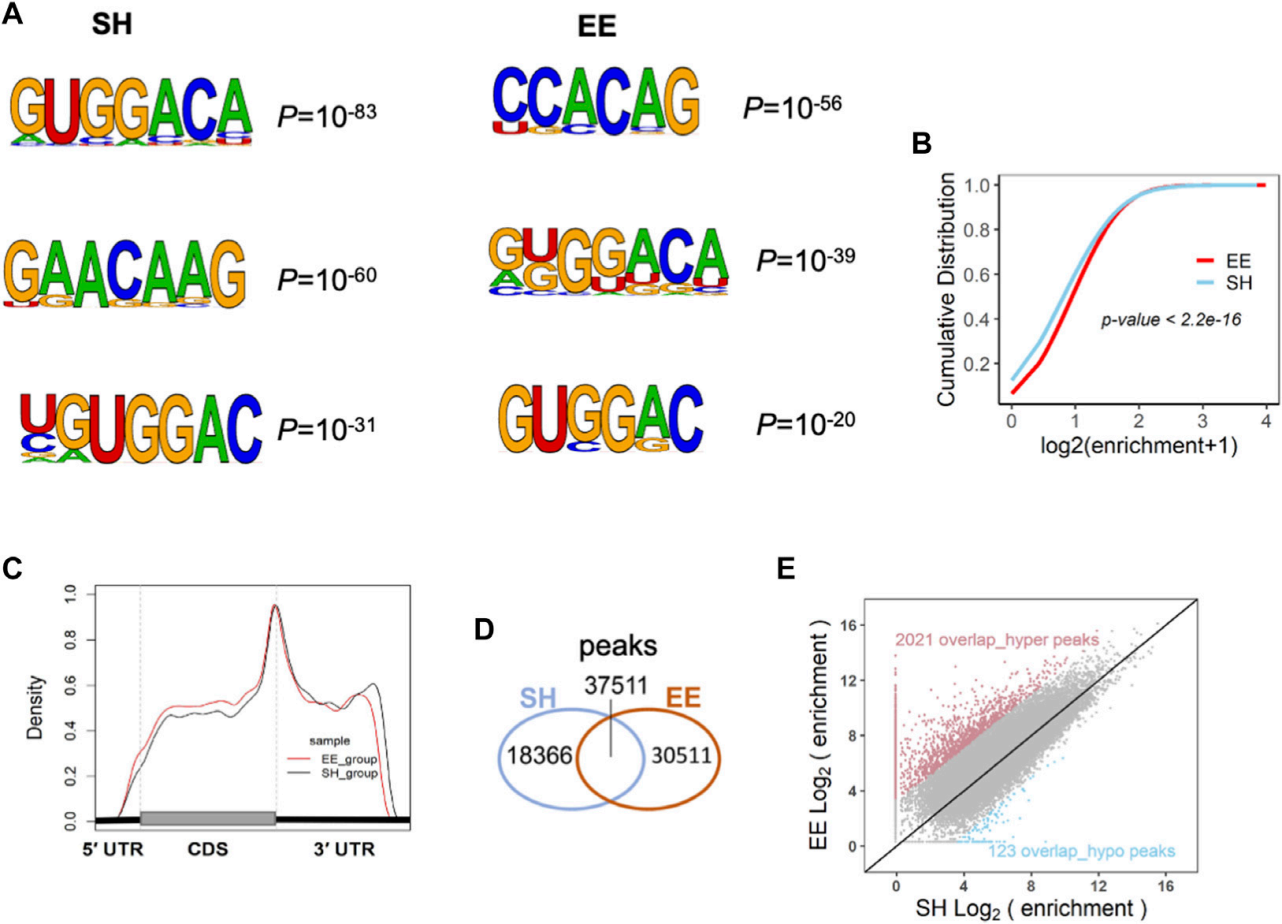
Enriched environment exposure alters the transcriptome-wide distribution of m^6^A. **(A)** MeRIP-seq data analysis revealed the sequence motif of m^6^A modification in SH and EE groups. **(B)** Cumulative distribution of m^6^A abundance (log_2_(enrichment folds+1)) in SH (blue line) and EE (red line) groups. *p* value was calculated using the two-sided Wilcoxon and Mann–Whitney test. **(C)** The distribution pattern of m^6^A modification in the transcriptome of the hippocampal tissues of SH and EE mice. m^6^A tag was significantly enriched around the stop codon of mRNAs. **(D)** Venn diagram illustrating the specific and overlapped m^6^A peaks in the transcriptome of the hippocampal tissues of SH and EE mice. **(E)** Scatterplot showing the differential m^6^A enrichment of the overlapped peaks between SH and EE groups, including 2021 hyper-methylated peaks (red dots) and 123 hypo-methylated peaks (blue dots) in the EE group.

The dynamic m^6^A methylation has been shown to be involved in the regulation of neuronal development, neurogenesis, and cognition (Meyer et al., 2012; Shafik et al., 2021; Cao et al., 2020; Li et al., 2018; Chen et al., 2019; Shi et al., 2018; Wang et al., 2018a; Hess et al., 2013; Nainar et al., 2016; Yoon et al., 2017; Livneh et al., 2020; Yu et al., 2017). To determine whether m^6^A modification is involved in the regulation of EE exposure in neurogenesis and neuronal development, we performed RNA m^6^A MeRIP-seq with SH and EE-treated mouse brain samples. The analysis of MeRIP-seq data of SH and EE groups revealed m^6^A motifs, which included canonical and novel m^6^A motifs (Figure 4A) (Batista et al., 2014; Chen et al., 2019). EE-treatment substantially increased m^6^A enrichment in amplitude compared to the SH group (Figure 4B). MeRIP-seq data analysis also showed that the enriched distribution of m^6^A peaks was close to stopping codons in both SH and EE groups (Figure 4C and Supplementary Figures S3A,B), whereas EE exposure increased the distribution of m^6^A methylation on coding sequence (CDS) compared to SH group (Figure 4C and Supplementary Figures S3A,B). Totals of 58,062 and 71,370 m^6^A peaks were identified in the SH group and EE group, respectively (Figure 4D and Supplementary Table S3). Among the identified peaks, 37,511 m^6^A peaks were overlapped between SH and EE groups; 18,366 m^6^A peaks were specific in the SH group and 30,511 m^6^A peaks were specific in the EE group, respectively (Figure 4D and Supplementary Table S3). The hypermethylated m^6^A peaks are predominantly localized at 3′ untranslated region (3′UTR) and coding sequence (CDS), whereas the hypomethylated peaks are predominantly localized at 3′UTR (Supplementary Figure S3C). As to the 37,511 overlapped m^6^A peaks, 2021 overlap_hyper- and 123 overlap_hypo-methylated m^6^A peaks were identified in the EE group compared to the SH group, respectively (Figure 4E and Supplementary Table S3).

We examined the differential m^6^A peaks identified in our study with the m^6^A sites included in the m^6^A atlas database, which collected 442,162 reliable m^6^A sites (Tang et al., 2021). Among 32,532 hyper_m^6^A peaks, 4309 peaks were mapped to 8856 m^6^A sites, which were correlated to 3541 genes, and 28,223 peaks were unmapped. Among the total of 18,489 hypo_m^6^A peaks, 1592 peaks were mapped to 3024 m^6^A sites, which were correlated to 1541 genes, and 16,897 peaks were unmapped (Supplementary Figures S3D,E). GO analysis showed that genes correlating to hyper_m^6^A peaks-mapped sites enriched terms for neuronal development, and genes correlating to hypo_m^6^A peaks-mapped sites enriched terms for cell cycle (Supplementary Figures S3F,G). We also analyzed the conservation of m^6^A peaks identified in our study by mapping to the ConsRM database (Song et al., 2021). SH and EE total m^6^A peaks were mapped to 11,058 and 11,464 ConsRM m^6^A sites, respectively (Supplementary Figures S3H,I). In addition, hyper_m^6^A peaks were mapped to 1859 ConsRM m^6^A sites, which were correlated to 1042 genes and hypo_m^6^A peaks were mapped to 595 ConsRM m^6^A sites, which were correlated to 389 genes, respectively (Supplementary Figure S3J). GO analysis showed that genes correlating with hyper_m^6^A peaks mapped m^6^A sites enriched terms for neuronal development, and genes correlating to hypo_m^6^A peaks-mapped sites enriched terms for cell cycle (Supplementary Figures S3K,L). These results suggest that EE exposure induces differential m^6^A modification in the brain, which could involve regulating neuronal function.

### Differentially Expressed Transcripts With Differential m^6^A Modification Involves in Neuronal Development

**FIGURE 5:**
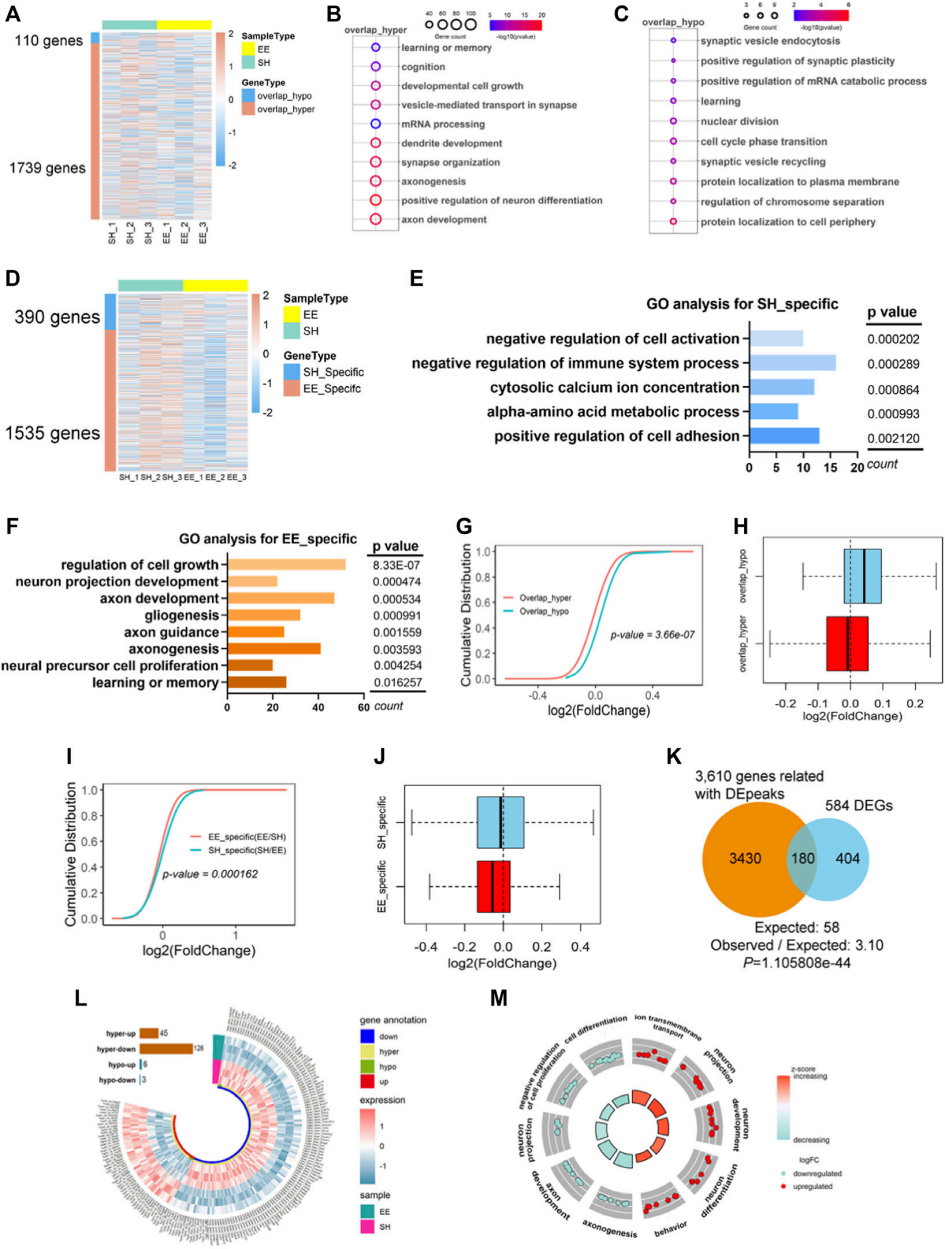
Differentially expressed transcripts with differential m^6^A modification involve in neuronal development. **(A)** Heatmap illustrating the 110 overlapped hypo-m^6^A (overlap_hypo) and 1739 hyper-m^6^A (overlap_hyper) peaks associated genes respectively. Three biological repeating hippocampal samples of SH and EE mice were adopted, respectively. **(B**,**C)** GO analysis shows that the functional enrichment of overlap_hyper- **(B)** and overlap_hypo-peaks **(C)** associated genes enriched learning and memory, synaptic development, and axon development. **(D)** Heatmap illustrating the 390 genes related to SH specific m^6^A peaks and 1535 genes related to EE specific m^6^A peaks. Three biological repeating hippocampal samples of SH and EE mice were adopted, respectively. **(E)** Top GO biological terms of specific m^6^A peaks associated genes in the SH group. Results show these genes were enriched in negative regulation of cell activation, metabolic process, and cell adhesion. **(F)** Top GO biological terms of specific m^6^A peaks associated genes in EE group. Results show these genes were enriched in the regulation of cell growth, neuronal development, axon development, and learning. **(G**,**H)** Cumulative curves **(G)** and box plot **(H)** of log_2_(fold change of gene expression). The red line and box plot show the expression of overlap_hyper-m^6^A methylation peaks associated with genes in SH and EE groups. The greenline and box plot show the expression of overlap_hypo-m^6^A methylation peaks associated with genes in SH and EE groups. **(I**,**J)** Cumulative curves **(I)** and box plot **(J)** of log_2_(fold change of gene expression). The green line and box plot shows SH-specific m^6^A peaks associated with genes. Redline and box plot show EE-specific m^6^A peaks associated with genes. **(K)** Venn diagram showing the overlapped genes between differential m^6^A peaks (including overlap_hyper, overlap_hypo, and SH/EE specific peaks) associated genes and differentially expressed genes (DEGs). *p* values were calculated by a hypergeometric test. **(L)** Circos plot showing the distribution of hyper-up, hyper-down, hypo-up, and hypo-down associated genes in EE group compared to SH group. hyper, including EE specific and overlap_hyper m^6^A peaks in EE group; hypo, including SH specific and overlap_hypo m^6^A peaks in EE group. **(M)** GO Circle visualization of 10 of the most relevant functional categories related to neurodevelopment and learning (five hyper-up regulated and five hyper-down regulated) in the EE group versus SH. The outer circle shows the log2 fold change (FC) of the genes in each category, the height of the inner bar plot indicates the significance level of the GO term (−log10(FDR)), and the color represents the Z score.

To examine the effects of EE exposure-induced the altered m^6^A modification on gene expression, we next performed the integrated analysis with MeRIP-seq data and RNA-seq data. As to the 37,511 common m^6^A peaks identified in both SH and EE groups, 123 overlap_hypo- and 2021 overlap_hyper-methylated m^6^A peaks were related to 119 and 1748 genes, respectively (Figure 5A and Supplementary Table S4). GO analysis revealed that both overlap_hyper and overlap_hypo m^6^A peaks-related genes were significantly enriched in biological processes relating to neuronal development and function, such as learning or memory, synaptic organization and plasticity, mRNA processing, *etc* (Figures 5B,C).

Next, we correlated specific m^6^A peaks with gene expression in SH and EE groups, respectively. We observed that the identified specific m^6^A peaks corresponded to 390 and 1535 genes in the SH group and EE group, respectively (Figure 5D and Supplementary Table S5). GO analysis showed that EE-specific m^6^A peaks-associated genes were enriched for regulation of cell growth, axon development, gliogenesis, *etc*, whereas SH-specific m^6^A peaks-associated genes were enriched for terms including negative regulation of cell activation and negative regulation of immune system process (Figures 5E,F). We observed that overlapped hypo-m^6^A modification-associated genes showed higher expression compared to that of hyper-m^6^A modification-associated genes (Figures 5G,H). Consistently, specific m^6^A modification was correlated with the decreased gene expression in both SH and EE groups (Figures 5I,J).

We next combined the gene lists with differential peaks, including specific peaks and overlapped_hyper and overlapped_hypo peaks, and harvested 3610 genes; 180 of these 3610 genes (4.99%) were overlapped with differential expressed genes (DEGs) (584) revealed by RNA-seq (30.82%) (Figure 5K and Supplementary Table S6). Forty-five genes with hyper m^6^A modification were up-regulated (hyper_up), and 126 genes with hyper m^6^A modification were down-regulated (hyper_down) (Figure 5L and Supplementary Table S6). Six genes with hypo m^6^A modification displayed increased expression, and three with hyper m^6^A modification displayed decreased expression (Figure 5L and Supplementary Table S6). Furthermore, GO analysis revealed that both hyper_up and hyper_down genes enriched for the terms including neuronal projection, neuron development, cell differentiation, and so on (Figure 5M). Together, these results suggest that differential m^6^A modification is involved in regulating the expression of genes related to neuronal development and neurogenesis.

### Fto Involves in EE Exposure-induced Differential m^6^A Modification

**FIGURE 6:**
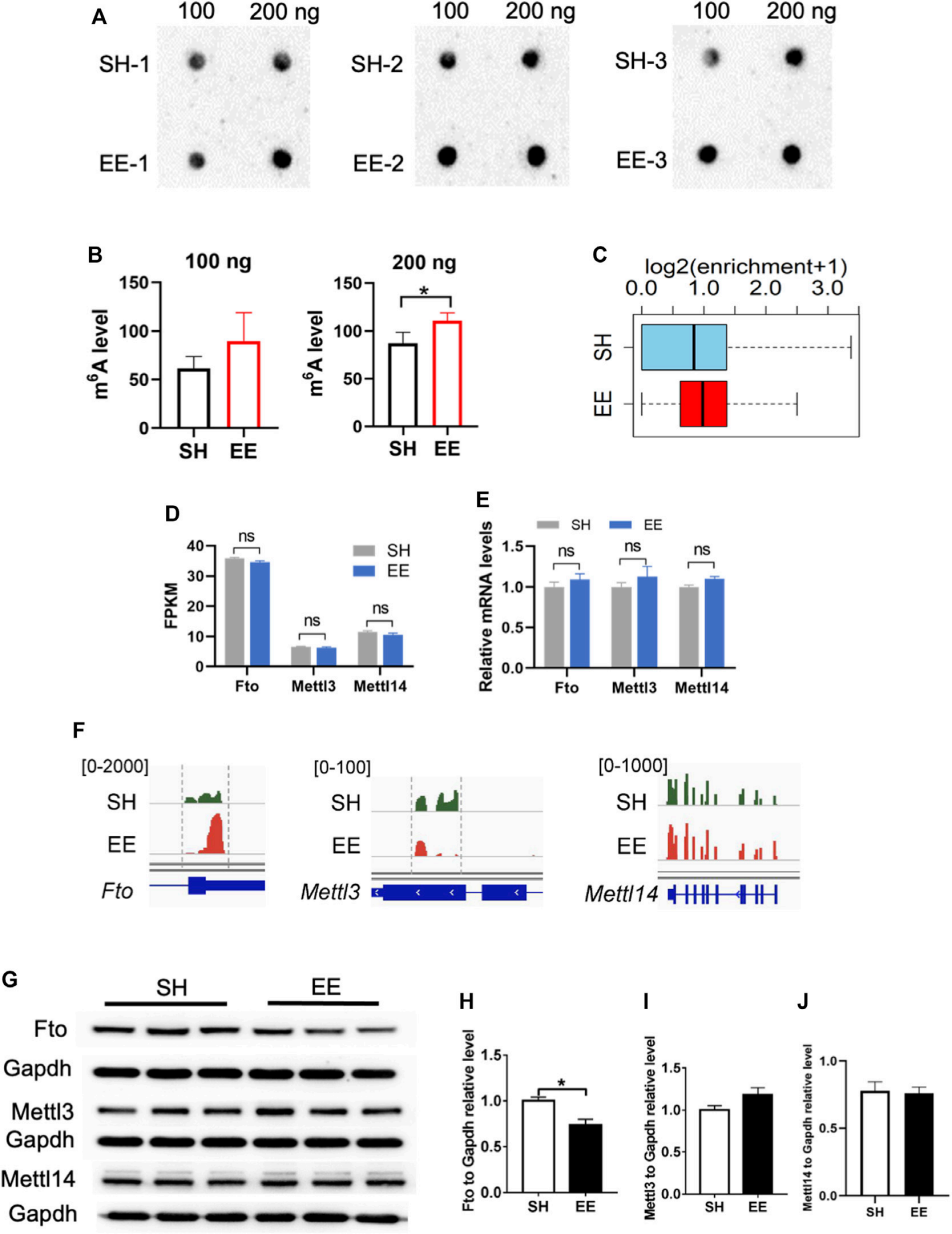
Fto involves in EE exposure-induced differential m^6^A modification. **(A**,**B)** m^6^A dot blot and quantification results showed that EE exposure induced a decrease of global m^6^A level compared to that of SH mice. *n* = 3 independent experiments. Values represent mean ± SEM; *p < 0.05; **p < 0.01; ***p < 0.001; unpaired Students t test. **(C)** Box plot of (log_2_(enrichment +1)) in SH (blue box) and EE (red box) groups. *p* value was calculated using two-sided Wilcoxon and Mann–Whitney test. **(D)** FPKM values of m^6^A writers *Mettl3*, *Mettl14*, and *Fto*. Values represent mean ± SEM; ns, not significant; *p < 0.05; **p < 0.01; ***p < 0.001; unpaired Students t test. **(E)** qRT-PCR results showing the expression of m^6^A writers *Mettl3*, *Mettl14*, and *Fto*. *n* = 3 independent experiments. Values represent mean ± SEM; ns, not significant; *p < 0.05; **p < 0.01; ***p < 0.001; unpaired Students t test. **(F)** IGV screenshots showing the altered m^6^A modification on Fto, Mettl3 and Mettl14 hypermethylation in EE compared to SH in the 3′ UTR of *Fto*. **(G**–**J)** Representative Western blot images **(G)** and quantification results **(H**–**J)** showed that EE exposure led to a decrease of FTO protein, whereas the levels of METTL3 and METTL14 were not changed. *n* = 3 independent experiments. Values represent mean ± SEM; ns, not significant; *p < 0.05; **p < 0.01; ***p < 0.001; unpaired Students t test.

Next, we performed an m^6^A dot blot and compared the levels of total m^6^A levels between SH and EE-treated mice. m^6^A dot blot assay and data analysis showed that the global level of m^6^A increased in the hippocampal tissues of EE exposed mice compared to that of SH mice (Figures 6A,B). In addition, MeRIP data analysis also revealed an increase in m^6^A modification in EE exposed mice compared to SH mice (Figure 6C).

Next, we examined the expression of several key genes related to m^6^A modification including Fto, Mettl3, and Mettl14. Although the expression of *Fto*, *Mettl3*, *Mettl14*, *Wtap*, *Rbm15,* and *Rbm15b* did not show significant differences at the mRNA level (Figures 6D,E, and Supplementary Figure S4A), *Fto* and *Mettl3* displayed differential m^6^A modification (Figure 6F). Western blot assay and data analysis showed that the protein level of Fto significantly decreased in EE exposed mice compared to that of SH mice (Figures 6G,H), but we did not observe a significant change in the levels of METTL3 and METTL14 (Figures 6G,I,J). These results together suggest that the Fto mediated the differential m^6^A mRNA modification induced by EE exposure.

## Discussion

In the present study, we investigated the effects of the enriched environment (EE) on neurogenesis and its related mechanisms during postnatal neuronal development. We found that EE exposure enhanced neurogenesis and cognition of mice. RNA sequencing revealed that EE exposure altered the transcriptome, which was related to neurogenesis, neuronal development, and so on. EE exposure altered the global level of mRNA m^6^A and several novel m^6^A motifs were uncovered besides canonical motifs. In addition, EE exposure induced differential m^6^A mRNA modification. Only 21.8% of differential peaks were mapped to the m^6^A atlas database, which included some “known m^6^A sites” (Tang et al., 2021), and around half of the differential peaks (49.5% in the SH group and 51.3% in EE group) were mapped to ConsRM database (Song et al., 2021). Genes correlated by these sites are involved in axon development and the cell cycle. These results suggest that epitranscriptome respond to environmental stimuli novel m^6^A sites could involve in the altered transcriptome during the postnatal neuronal development. Genetic manipulation of m^6^A writers, erasers, and readers affected m^6^A methylation level and altered gene expression, which in turn regulated neuronal development and neurogenesis, and involves in neurological disease (Hess et al., 2013; Batista et al., 2014; Wang et al., 2014; Sevgi et al., 2015; Nainar et al., 2016; Widagdo et al., 2016; Bartosovic et al., 2017; Li et al., 2017b; Yu et al., 2017; Wang et al., 2018a; Engel et al., 2018; Chen et al., 2019; Du et al., 2019; Cao et al., 2020; Gao et al., 2020; Livneh et al., 2020; Shafik et al., 2021). m^6^A methylation plays the regulatory role of gene expression at transcriptive and translational levels (Fu et al., 2014; Aguilo et al., 2015; Chen et al., 2016; Yang et al., 2018; Chen et al., 2019). Our present study also showed that more than one-third of differentially (including up- and down-regulated) expressed genes displayed differential m^6^A methylation induced by EE exposure. An enriched environment promotes neurogenesis and improves the learning of adult and aged animals (Kempermann et al., 1997; Nilsson et al., 1999; van Praag et al., 2005; Kempermann, 2019; Gronska-Peski et al., 2021). In addition, environmental stimuli induce structural changes in the brain, promote the development of behavioral variability, and generate persistent individualized behavior in mice (Freund et al., 2013; Kempermann, 2019; Zocher et al., 2020). One of the underlying mechanisms is the interaction between environmental stimuli and epigenetics.

Enriched environment exposure displays effects on DNA methylation (5-methylcytosine, 5mC), 5-hydroxymethylation (5-hydroxymethylcytosine, 5hmC), and histone modifications (Fischer et al., 2007; Irier et al., 2014; Zocher et al., 2021). Our present results showed that an enriched environment not only affects the global level of mRNA m^6^A but also induces the differential m^6^A mRNA modification. Previous studies had shown that both m^6^A writers and erasers play an important function in the neuronal system (Hess et al., 2013; Sevgi et al., 2015; Nainar et al., 2016; Widagdo et al., 2016; Li et al., 2017b; Wang et al., 2018a; Shi et al., 2018; Weng et al., 2018; Chen et al., 2019; Cao et al., 2020; Gao et al., 2020), however, our present data showed that EE exposure display significant effects on the expression of m^6^A eraser FTO, but not the key m^6^A writers METTL3 and METTL14. Although the underlying mechanism is unclear, we speculate that EE exposure induced more activity in animals, which could affect the metabolic state of animals, and consequently alter the expression of FTO. In summary, our results suggest an effect of an enriched environment on mRNA m^6^A modification and add a novel layer for the interaction between environment and epigenetics in the context of neuronal development.

## Materials and Methods

### Animals and Enriched Environment

Male C57BL/6 mouse pups were weaned at the age of 3-week, randomly grouped, and housed in the standard cage (SH) and enriched environment (EE), respectively (Supplementary Figure S1A). As to the EE housing, the cage was equipped with plastic toys, tunnels, and hideouts, which were replaced and rearranged every week. Five weeks later (at the age of postnatal 8-week), behavioral tests and multiple assays were performed to analyze the effects of EE exposure.

Mice were housed in the laboratory animal center of Zhejiang University under 12-h light/12-h dark conditions with free access to food and water. All animal experiments were carried out following the protocols approved by the Zhejiang University Animal Care and Use Committee.

### BrdU Administration and Preparation of Brain Sections

Animals were injected intraperitoneally (i.p.) with BrdU (50 mg/kg) at the age of 8-week, six times in 24 h with an interval of 4 h (proliferation assay), and two times per day with an interval of 12 h for three consecutive days (differentiation assay) (Supplementary Figures S1B,C). Animals were sacrificed at 1 day, 1 or 4 weeks (s) post the final BrdU injection, respectively (Supplementary Figures S1B,C). At the scheduled time point, mice were transcardially perfused with ice-cold phosphate-buffered saline (PBS) followed by 4% paraformaldehyde and complete dehydration with 30% sucrose at 4°C. Coronal sections were cut with a cryostat (Leica, CM 1950) and collected into the cryoprotectant solution. Sections were stored in cryoprotectant solution at −20°C until further processing.

### Immunofluorescence Staining and Cell Quantification

For immunostaining, every one in six serial coronal sections containing the hippocampal region were picked up and six to eight sections of each animal were adopted for the assay. After being washed with PBS following the treatment with a blocking solution containing 3% normal goat serum and 0.1% Triton X-100 in PBS for 1 h at room temperature, sections were incubated with primary antibodies overnight at 4°C. For BrdU immunostaining, samples were pretreated with 1 M HCl at 37°C for 30 min before blocking. On the second day, sections were incubated with fluorescent secondary antibodies for 1 h at room temperature. After washing, samples were mounted onto glass slides and images were captured using a Nikon inverted microscope. The numbers of BrdU^+^, BrdU^+^DCX^+,^ and BrdU^+^NeuN^+^ cells were quantified. The representative images were captured with an Olympus FLUOVIEW FV3000 confocal microscope. The following primary antibodies were used: anti-BrdU (Catalog# ab6326; Abcam), anti-DCX (Catalog# 4604; Cell Signaling Technology), and anti-NeuN (Catalog# MAB377; Millipore). The used secondary antibody included AlexaFluor568 goat anti-rat (Cat# A11077; Thermo Fisher Scientific), AlexaFluor488 goat anti-mouse (Catalog# A11001; Thermo Fisher Scientific), and AlexaFluor568 goat anti-rabbit (Cat#A11036; Thermo Fisher Scientific).

### Western Blot

Tissue samples were homogenized in RIPA lysis buffer (Catalog# ab156034; Abcam) containing 1X protease inhibitor cocktail (Catalog# 04693124001; Sigma). Protein of 20 μg was subjected to SDS-PAGE separation, and the membrane was incubated with primary antibodies overnight at 4°C. The used primary antibodies included anti-METTL3 (Catalog# 21207-1-AP; Proteintech), anti-METTL14 (Catalog# HPA038002; ATLAS), anti-FTO (Catalog# PA1-46310; Thermo Fisher Scientific) and anti-GAPDH (Catalog# AM4300; Thermo Fisher Scientific). On the second day, HRP conjugated secondary antibodies were applied for 1 h at room temperature. The signal was detected by Tanon 5200 detection system (Shanghai, China). The intensity of immunoblot bands was normalized to Gapdh and analyzed with ImageJ software.

### RNA Isolation and m^6^A Dot-Blot Assay

Total RNA was extracted with TRIzol reagent (Catalog# 1559 ‘6018; Thermo Fisher Scientific) following the manufacturer’s protocol. For the m^6^A dot-blot assay, total RNA samples were denatured at 65°C and then spotted onto Hybond N+ membranes (Catalog# NP1096; GE Healthcare). Membranes were blocked with 5% milk for 1 h at room temperature followed by the incubation with an anti-m^6^A antibody (Catalog# 202003; Synaptic Systems) overnight at 4°C. On the second day, HRP-conjugated secondary antibody was applied for 30 min at room temperature. The signal was detected Tanon 5200 detection system and analyzed with ImageJ software.

### RNA-Seq and Data Analysis

Total RNA concentration was determined spectrophotometrically using NanoDrop (NanoDrop Technologies). RNA integrity value (RIN) was determined with the RNA Nano 6000 Assay Kit of the Bioanalyzer 2100 system (Agilent Technologies Inc.). Sequencing libraries were generated and index codes were added to attribute sequences to each sample using NEBNext^®^ UltraTM RNA Library Prep Kit for Illumina^®^ (NEB).

For RNA-seq data analysis, raw reads of fastq format were processed and clean reads were generated by removing reads containing adapter and ploy-N from raw data. Reads were mapped to the mouse genome (mm10) with Hisat2 by the default parameters (Kim et al., 2015). The read numbers mapped to each gene were counted with FeatureCounts 1.5.0-p3. Fragments per kilobase of exon per million mapped reads (FPKM) of each gene were calculated based on the length of the gene and reads mapped to this gene. Differentially expressed genes (DEGs) between samples were analyzed with edgeR. *p*-Value of 0.01 and absolute fold change of 1 were set as the threshold for differential significance of expression.

### MeRIP-Seq Data Analysis

MeRIP-seq data analysis was performed as described previously (Chen et al., 2019). Briefly, adaptor sequences and bases with low quality were removed from raw reads with Trimmomatic software (Bolger et al., 2014). The clean reads were then aligned to the mouse genome (mm10) using Hisat2 with default parameters (Kim et al., 2019). Only unique mapped reads with mapping quality ≥30 were adopted for the subsequent analysis.

For MeRIP-seq peak calling, MACS2 (version 2.2.7) was run with the default options except for “-p 0.05 -nomodel, -keepdup all” to turn off fragment size estimation and to keep all uniquely mapping reads with the corresponding input sample serving as control, respectively (Zhang et al., 2008). To identify specific or overlapped m^6^A peaks, peaks were intersected using the BedTools package which overlapped peaks were set up “-f 0.5” (Quinlan and Hall, 2010). DiffBind packages were used to identify differential enrichment overlapped peaks (Kolodziej-Wojnar et al., 2020). The differential peaks were determined with log2 (fold change) >1 or log2 (fold change) < −1 and *p* value <0.05. Motifs enriched with m^6^A peaks within all mRNAs were identified using HOMER software (v4.10) (Heinz et al., 2010). The motif length was restricted to five to six nucleotides.

### Functional Enrichment Analysis

Gene Ontology (GO) enrichment analysis was performed with clusterProfiler R package and DAVID database (Dennis et al., 2003). GO terms with corrected *p*-value less than 0.05 were considered significant for the enrichment of differentially expressed genes.

### Behavioral Tests

The pattern separation test was performed as described previously (Tracy et al., 2016). The test consists of a sample phase and a test phase. During the sample phase, animals were placed in an open chamber with two identical objects (context A) and allowed to freely explore for 5 min. After a 30-min interval, mice were placed in another open chamber (context B) with two identical objects that were different from those in context A. After 4 h, one of the objects in both open chambers was exchanged. The time mice explored incongruent object that was from another context in the sample phase was compared with the time mice explored the congruent object.

The Morris water maze (MWM) test was carried out as described previously (Li et al., 2017a). During the training stage, adult (9-week-old) male mice were placed in the water maze from four different starting locations (NE, NW, SE, and SW), respectively. Each animal would execute four trials per day for four consecutive days. On the fifth day, the platform was removed and the time of swimming in each quadrant was measured over 60 s for a probe trial. All trials were videotaped and the data were analyzed with MazeScan software (Actimetrics, China).

For the eight-arm radial maze test, adult male mice were fasting for 24 h before training to adjust their bodyweight to 80–85% of free-eating animals (Chen et al., 2021). On the first day, four mice in groups were kept in the full-baited maze in which baits (chocolate particles) were scattered in the center and the end of the arms, and animals could explore each arm of the maze freely for 10 min. On the second and third days, a maze with a bar of chocolate was placed only at the end of each arm, and an individual mouse was placed on the center platform for 10 s and allowed to move freely for 10 min or until all chocolate pellets were consumed. For the next days, only four of eight arms were baited, and food rewards were placed at the end of the same four arms during this session. Mice were allowed to move freely until all chocolate pellets were consumed, otherwise, the trial was terminated at the time point of 10 min. Working memory error was counted when animals traveled to an arm that was already visited, and reference memory error was counted when animals traveled to a non-baited arm with all four paws. The maze was cleaned with 70% ethanol after each animal completed the test. The test was videotaped and the data were analyzed with MazeScan software (Med Associates Inc).

### Statistical Analysis

Data were presented as mean ± SEM. Statistical analysis between-group differences was performed with a two-tail unpaired Student’s t-test using Graph Prism software (version 9.0, GraphPad). *p* < 0.05 was considered statistically significant. For multiple group comparisons, a two-way ANOVA followed by Tukey’s *post hoc* test was used. Statistical significance was defined as a *p*-value <0.05. Replicate information is indicated in the figure legends.

## Data Availability

The datasets presented in this study can be found in online repositories. The names of the repository/repositories and accession number(s) can be found below: Gene Expression Omnibus accession number: GSE197754.

## References

[B1] Aguilo F. Zhang F. Sancho A. Fidalgo M. Di Cecilia S. Vashisht A. (2015). Coordination of M 6 A mRNA Methylation and Gene Transcription by ZFP217 Regulates Pluripotency and Reprogramming. Cell Stem Cell 17, 689–704. 10.1016/j.stem.2015.09.005 26526723 PMC4671830

[B2] Atlasi Y. Stunnenberg H. G. (2017). The Interplay of Epigenetic Marks during Stem Cell Differentiation and Development. Nat. Rev. Genet. 18, 643–658. 10.1038/nrg.2017.57 28804139

[B3] Avgustinova A. Benitah S. A. (2016). Epigenetic Control of Adult Stem Cell Function. Nat. Rev. Mol. Cell Biol. 17, 643–658. 10.1038/nrm.2016.76 27405257

[B4] Bartosovic M. Molares H. C. Gregorova P. Hrossova D. Kudla G. Vanacova S. (2017). N6-methyladenosine Demethylase FTO Targets Pre-mRNAs and Regulates Alternative Splicing and 3'-end Processing. Nucleic Acids Res. 45, 11356–11370. 10.1093/nar/gkx778 28977517 PMC5737695

[B5] Batista P. J. Molinie B. Wang J. Qu K. Zhang J. Li L. (2014). m6A RNA Modification Controls Cell Fate Transition in Mammalian Embryonic Stem CellsA RNA Modification Controls Cell Fate Transition in Mammalian Embryonic Stem Cells. Cell Stem Cell 15, 707–719. 10.1016/j.stem.2014.09.019 25456834 PMC4278749

[B6] Cao Y. Zhuang Y. Chen J. Xu W. Shou Y. Huang X. (2020). Dynamic Effects of Fto in Regulating the Proliferation and Differentiation of Adult Neural Stem Cells of Mice. Hum. Mol. Genet. 29, 727–735. 10.1093/hmg/ddz274 31751468

[B7] Chen J. Dong X. Cheng X. Zhu Q. Zhang J. Li Q. (2021). Ogt Controls Neural Stem/progenitor Cell Pool and Adult Neurogenesis through Modulating Notch Signaling. Cell Rep. 34, 108905. 10.1016/j.celrep.2021.108905 33789105

[B8] Chen J. Zhang Y.-C. Huang C. Shen H. Sun B. Cheng X. (2019). m6A Regulates Neurogenesis and Neuronal Development by Modulating Histone Methyltransferase Ezh2A Regulates Neurogenesis and Neuronal Development by Modulating Histone Methyltransferase Ezh2. Genomics, proteomics Bioinforma. 17, 154–168. 10.1016/j.gpb.2018.12.007 PMC6620265 31154015

[B9] Chen K. Zhao B. S. He C. (2016). Nucleic Acid Modifications in Regulation of Gene Expression. Cell Chem. Biol. 23, 74–85. 10.1016/j.chembiol.2015.11.007 26933737 PMC4779186

[B10] DennisG.Jr.ShermanB. T.HosackD. A.YangJ.GaoW.LaneH. C. (2003). DAVID: Database for Annotation, Visualization, and Integrated Discovery. Genome Biol. 4, P3. 10.1186/gb-2003-4-5-p3 12734009

[B11] Du K. Zhang L. Lee T. Sun T. (2019). m6A RNA Methylation Controls Neural Development and Is Involved in Human DiseasesA RNA Methylation Controls Neural Development and Is Involved in Human Diseases. Mol. Neurobiol. 56, 1596–1606. 10.1007/s12035-018-1138-1 29909453

[B12] Engel M. Eggert C. Kaplick P. M. Eder M. Röh S. Tietze L. (2018). The Role of m6A/m-RNA Methylation in Stress Response Regulation. Neuron 99, 389–403. 10.1016/j.neuron.2018.07.009 30048615 PMC6069762

[B13] Fischer A. Sananbenesi F. Wang X. Dobbin M. Tsai L.-H. (2007). Recovery of Learning and Memory Is Associated with Chromatin Remodelling. Nature 447, 178–182. 10.1038/nature05772 17468743

[B14] Freund J. Brandmaier A. M. Lewejohann L. Kirste I. Kritzler M. Kruger A. (2013). Emergence of Individuality in Genetically Identical Mice. Science 340, 756–759. 10.1126/science.1235294 23661762

[B15] Fu Y. Dominissini D. Rechavi G. He C. (2014). Gene Expression Regulation Mediated through Reversible m6A RNA Methylation. Nat. Rev. Genet. 15, 293–306. 10.1038/nrg3724 24662220

[B16] Fustin J.-M. Doi M. Yamaguchi Y. Hida H. Nishimura S. Yoshida M. (2013). RNA-methylation-dependent RNA Processing Controls the Speed of the Circadian Clock. Cell 155, 793–806. 10.1016/j.cell.2013.10.026 24209618

[B17] Gao H. Cheng X. Chen J. Ji C. Guo H. Qu W. (2020). Fto-modulated Lipid Niche Regulates Adult Neurogenesis through Modulating Adenosine Metabolism. Hum. Mol. Genet. 29, 2775–2787. 10.1093/hmg/ddaa171 32766784

[B18] Gronska-Peski M. Goncalves J. T. Hebert J. M. (2021). Enriched Environment Promotes Adult Hippocampal Neurogenesis through FGFRs. J. Neurosci. 41, 2899–2910. 33637561 10.1523/JNEUROSCI.2286-20.2021 PMC8018882

[B19] Hess M. E. Hess S. Meyer K. D. Verhagen L. A. W. Koch L. Brönneke H. S. (2013). The Fat Mass and Obesity Associated Gene (Fto) Regulates Activity of the Dopaminergic Midbrain Circuitry. Nat. Neurosci. 16, 1042–1048. 10.1038/nn.3449 23817550

[B20] Hsieh J. Zhao X. (2016). Genetics and Epigenetics in Adult Neurogenesis. Cold Spring Harb. Perspect. Biol. 8. 10.1101/cshperspect.a018911 PMC4888816 27143699

[B21] Irier H. Street R. C. Dave R. Lin L. Cai C. Davis T. H. (2014). Environmental Enrichment Modulates 5-hydroxymethylcytosine Dynamics in hippocampus. Genomics 104, 376–382. 10.1016/j.ygeno.2014.08.019 25205305 PMC4252786

[B22] Kempermann G. (2019). Environmental Enrichment, New Neurons and the Neurobiology of Individuality. Nat. Rev. Neurosci. 20, 235–245. 10.1038/s41583-019-0120-x 30723309

[B23] Kempermann G. Kuhn H. G. Gage F. H. (1997). More Hippocampal Neurons in Adult Mice Living in an Enriched Environment. Nature 386, 493–495. 10.1038/386493a0 9087407

[B24] Kim D. Langmead B. Salzberg S. L. (2015). HISAT: a Fast Spliced Aligner with Low Memory Requirements. Nat. Methods 12, 357–360. 10.1038/nmeth.3317 25751142 PMC4655817

[B25] Kim D. Paggi J. M. Park C. Bennett C. Salzberg S. L. (2019). Graph-based Genome Alignment and Genotyping with HISAT2 and HISAT-Genotype. Nat. Biotechnol. 37, 907–915. 10.1038/s41587-019-0201-4 31375807 PMC7605509

[B26] Li H.-B. Tong J. Zhu S. Batista P. J. Duffy E. E. Zhao J. (2017). m6A mRNA Methylation Controls T Cell Homeostasis by Targeting the IL-7/STAT5/SOCS Pathways. Nature 548, 338–342. 10.1038/nature23450 28792938 PMC5729908

[B27] Li L. Zang L. Zhang F. Chen J. Shen H. Shu L. (2017). Fat Mass and Obesity-Associated (FTO) Protein Regulates Adult Neurogenesis. Hum. Mol. Genet. 26, 2398–2411. 10.1093/hmg/ddx128 28398475 PMC6192412

[B28] Li M. Zhao X. Wang W. Shi H. Pan Q. Lu Z. (2018). Ythdf2-mediated m6A mRNA Clearance Modulates Neural Development in Mice. Genome Biol. 19, 69. 10.1186/s13059-018-1436-y 29855337 PMC5984442

[B29] Li X. Jin P. (2010). Roles of Small Regulatory RNAs in Determining Neuronal Identity. Nat. Rev. Neurosci. 11, 329–338. 10.1038/nrn2739 20354535

[B30] Liu S. Xiu J. Zhu C. Meng K. Li C. Han R. (2021). Fat Mass and Obesity-Associated Protein Regulates RNA Methylation Associated with Depression-like Behavior in Mice. Nat. Commun. 12, 6937. 10.1038/s41467-021-27044-7 34836959 PMC8626436

[B31] Livneh I. Moshitch-Moshkovitz S. Amariglio N. Rechavi G. Dominissini D. (2020). The m6A Epitranscriptome: Transcriptome Plasticity in Brain Development and Function. Nat. Rev. Neurosci. 21, 36–51. 10.1038/s41583-019-0244-z 31804615

[B32] Lupori L. Cornuti S. Mazziotti R. Borghi E. Ottaviano E. Cas M. D. (2022). The Gut Microbiota of Environmentally Enriched Mice Regulates Visual Cortical Plasticity. Cell Rep. 38, 110212. 10.1016/j.celrep.2021.110212 35021093

[B33] Ma C. Chang M. Lv H. Zhang Z.-W. Zhang W. He X. (2018). RNA m6A Methylation Participates in Regulation of Postnatal Development of the Mouse Cerebellum. Genome Biol. 19, 68. 10.1186/s13059-018-1435-z 29855379 PMC5984455

[B34] Ma D. K. Marchetto M. C. Guo J. U. Ming G.-l. Gage F. H. Song H. (2010). Epigenetic Choreographers of Neurogenesis in the Adult Mammalian Brain. Nat. Neurosci. 13, 1338–1344. 10.1038/nn.2672 20975758 PMC3324277

[B35] Meyer K. D. Saletore Y. Zumbo P. Elemento O. Mason C. E. Jaffrey S. R. (2012). Comprehensive Analysis of mRNA Methylation Reveals Enrichment in 3′ UTRs and Near Stop Codons. Cell 149, 1635–1646. 10.1016/j.cell.2012.05.003 22608085 PMC3383396

[B36] Nainar S. Marshall P. R. Tyler C. R. Spitale R. C. Bredy T. W. (2016). Evolving Insights into RNA Modifications and Their Functional Diversity in the Brain. Nat. Neurosci. 19, 1292–1298. 10.1038/nn.4378 27669990 PMC5068363

[B37] Nilsson M. Perfilieva E. Johansson U. Orwar O. Eriksson P. S. (1999). Enriched Environment Increases Neurogenesis in the Adult Rat Dentate Gyrus and Improves Spatial Memory. J. Neurobiol. 39, 569–578. 10.1002/(sici)1097-4695(19990615)39:4<569::aid-neu10>3.0.co;2-f 10380078

[B38] Overall R. W. Paszkowski-Rogacz M. Kempermann G. (2012). The Mammalian Adult Neurogenesis Gene Ontology (MANGO) Provides a Structural Framework for Published Information on Genes Regulating Adult Hippocampal Neurogenesis. PLoS One 7, e48527. 10.1371/journal.pone.0048527 23139788 PMC3489671

[B39] Quinlan A. R. Hall I. M. (2010). BEDTools: a Flexible Suite of Utilities for Comparing Genomic Features. Bioinformatics 26, 841–842. 10.1093/bioinformatics/btq033 20110278 PMC2832824

[B40] Rampon C. Jiang C. H. Dong H. Tang Y.-P. Lockhart D. J. Schultz P. G. (2000). Effects of Environmental Enrichment on Gene Expression in the Brain. Proc. Natl. Acad. Sci. U.S.A. 97, 12880–12884. 10.1073/pnas.97.23.12880 11070096 PMC18858

[B41] Sevgi M. Rigoux L. Kuhn A. B. Mauer J. Schilbach L. Hess M. E. (2015). An Obesity-Predisposing Variant of the FTO Gene Regulates D2R-dependent Reward Learning. J. Neurosci. 35, 12584–12592. 10.1523/jneurosci.1589-15.2015 26354923 PMC6605390

[B42] Shafik A. M. Zhang F. Guo Z. Dai Q. Pajdzik K. Li Y. (2021). N6-methyladenosine Dynamics in Neurodevelopment and Aging, and its Potential Role in Alzheimer's Disease. Genome Biol. 22, 17. 10.1186/s13059-020-02249-z 33402207 PMC7786910

[B43] Shi H. Zhang X. Weng Y.-L. Lu Z. Liu Y. Lu Z. (2018). m6A Facilitates Hippocampus-dependent Learning and Memory through YTHDF1A Facilitates Hippocampus-dependent Learning and Memory through YTHDF1. Nature 563, 249–253. 10.1038/s41586-018-0666-1 30401835 PMC6226095

[B44] Song B. Chen K. Tang Y. Wei Z. Su J. de Magalhaes J. P. (2021). ConsRM: Collection and Large-Scale Prediction of the Evolutionarily Conserved RNA Methylation Sites, with Implications for the Functional Epitranscriptome. Briefings Bioinforma. 22. 10.1093/bib/bbab088 33993206

[B45] Sun W. Guan M. Li X. (2014). 5-hydroxymethylcytosine-mediated DNA Demethylation in Stem Cells and Development. Stem Cells Dev. 23, 923–930. 10.1089/scd.2013.0428 24400731

[B46] Tang Y. Chen K. Song B. Ma J. Wu X. Xu Q. (2021). m6A-Atlas: a Comprehensive Knowledgebase for Unraveling the N6-Methyladenosine (m6A) Epitranscriptome. Nucleic Acids Res. 49, D134–D143. 10.1093/nar/gkaa692 32821938 PMC7779050

[B47] Tracy T. E. Sohn P. D. Minami S. S. Wang C. Min S.-W. Li Y. (2016). Acetylated Tau Obstructs KIBRA-Mediated Signaling in Synaptic Plasticity and Promotes Tauopathy-Related Memory Loss. Neuron 90, 245–260. 10.1016/j.neuron.2016.03.005 27041503 PMC4859346

[B48] van Praag H. Shubert T. Zhao C. Gage F. H. (2005). Exercise Enhances Learning and Hippocampal Neurogenesis in Aged Mice. J. Neurosci. 25, 8680–8685. 10.1523/jneurosci.1731-05.2005 16177036 PMC1360197

[B49] Wang C.-X. Cui G.-S. Liu X. Xu K. Wang M. Zhang X.-X. (2018). METTL3-mediated m6A Modification Is Required for Cerebellar Development. PLoS Biol. 16, e2004880. 10.1371/journal.pbio.2004880 29879109 PMC6021109

[B50] Wang Y. Li Y. Toth J. I. Petroski M. D. Zhang Z. Zhao J. C. (2014). N6-methyladenosine Modification Destabilizes Developmental Regulators in Embryonic Stem Cells. Nat. Cell Biol. 16, 191–198. 10.1038/ncb2902 24394384 PMC4640932

[B51] Wang Y. Li Y. Yue M. Wang J. Kumar S. Wechsler-Reya R. J. (2018). N6-methyladenosine RNA Modification Regulates Embryonic Neural Stem Cell Self-Renewal through Histone Modifications. Nat. Neurosci. 21, 195–206. 10.1038/s41593-017-0057-1 29335608 PMC6317335

[B52] Weng Y.-L. Wang X. An R. Cassin J. Vissers C. Liu Y. (2018). Epitranscriptomic m6A Regulation of Axon Regeneration in the Adult Mammalian Nervous System. Neuron 97, 313–325. 10.1016/j.neuron.2017.12.036 29346752 PMC5777326

[B53] Widagdo J. Zhao Q.-Y. Kempen M.-J. Tan M. C. Ratnu V. S. Wei W. (2016). Experience-Dependent Accumulation of N 6 -Methyladenosine in the Prefrontal Cortex Is Associated with Memory Processes in Mice. J. Neurosci. 36, 6771–6777. 10.1523/jneurosci.4053-15.2016 27335407 PMC4916251

[B54] Yang Y. Hsu P. J. Chen Y. S. Yang Y. G. (2018). Dynamic Transcriptomic M(6)A Decoration: Writers, Erasers, Readers and Functions in RNA Metabolism. Cell Res. 28, 616–624. 10.1038/s41422-018-0040-8 29789545 PMC5993786

[B55] Yao B. Christian K. M. He C. Jin P. Ming G.-l. Song H. (2016). Epigenetic Mechanisms in Neurogenesis. Nat. Rev. Neurosci. 17, 537–549. 10.1038/nrn.2016.70 27334043 PMC5610421

[B56] Yoon K.-J. Ringeling F. R. Vissers C. Jacob F. Pokrass M. Jimenez-Cyrus D. (2017). Temporal Control of Mammalian Cortical Neurogenesis by m6A Methylation. Cell 171, 877–889. 10.1016/j.cell.2017.09.003 28965759 PMC5679435

[B57] Yu J. Chen M. Huang H. Zhu J. Song H. Zhu J. (2017). Dynamic m6A Modification Regulates Local Translation of mRNA in Axons. Nucleic Acids Res. 28, 616–624. 10.1093/nar/gkx1182 PMC5815124 29186567

[B58] Zhang C. Chen Y. Sun B. Wang L. Yang Y. Ma D. (2017). m6A Modulates Haematopoietic Stem and Progenitor Cell Specification. Nature 549, 273–276. 10.1038/nature23883 28869969

[B59] Zhang Y. Liu T. Meyer C. A. Eeckhoute J. Johnson D. S. Bernstein B. E. (2008). Model-based Analysis of ChIP-Seq (MACS). Genome Biol. 9, R137. 10.1186/gb-2008-9-9-r137 18798982 PMC2592715

[B60] Zhao F. Xu Y. Gao S. Qin L. Austria Q. Siedlak S. L. (2021). METTL3-dependent RNA m6A Dysregulation Contributes to Neurodegeneration in Alzheimer's Disease through Aberrant Cell Cycle Events. Mol. Neurodegener. 16, 70. 10.1186/s13024-021-00484-x 34593014 PMC8482683

[B61] Zhao X. Yang Y. Sun B.-F. Shi Y. Yang X. Xiao W. (2014). FTO-dependent Demethylation of N6-Methyladenosine Regulates mRNA Splicing and Is Required for Adipogenesis. Cell Res. 24, 1403–1419. 10.1038/cr.2014.151 25412662 PMC4260349

[B62] Zocher S. Schilling S. Grzyb A. N. Adusumilli V. S. Bogado Lopes J. Günther S. (2020). Early-life Environmental Enrichment Generates Persistent Individualized Behavior in Mice. Sci. Adv. 6, eabb1478. 10.1126/sciadv.abb1478 32923634 PMC7449688

[B63] Zocher S. Overall R. W. Lesche M. Dahl A. Kempermann G. (2021). Environmental Enrichment Preserves a Young DNA Methylation Landscape in the Aged Mouse hippocampus. Nat. Commun. 12, 3892. 10.1038/s41467-021-23993-1 34162876 PMC8222384

