# Supplementary figures and images for: m6A Modification Involves in Enriched Environment-Induced Neurogenesis and Cognition Enhancement
Source: Front Cell Dev Biol. 2022 Jun 2;10:903179. doi: 10.3389/fcell.2022.903179 (PMC9201454; doi:10.3389/fcell.2022.903179)

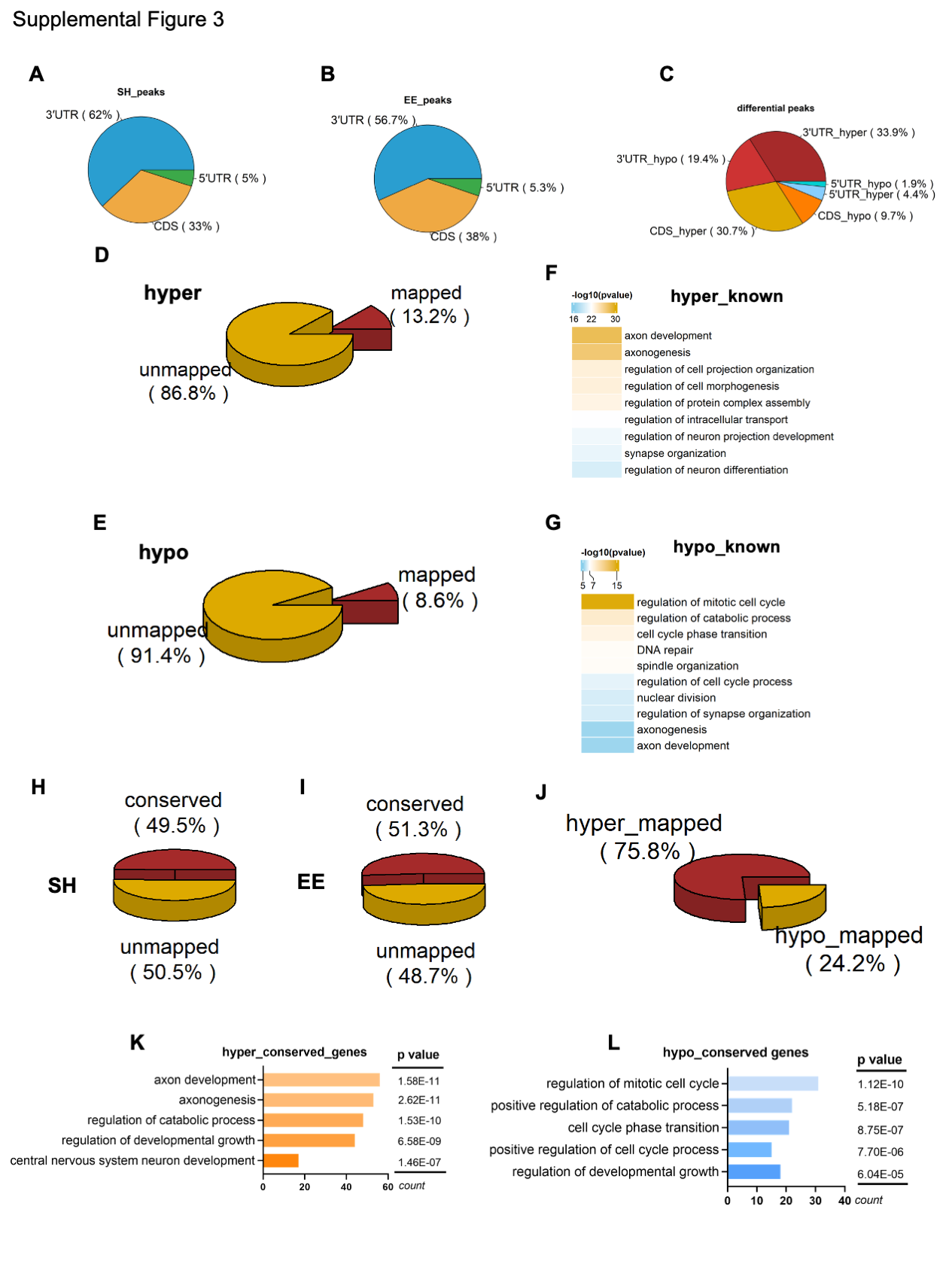

Supplement: Supplementary file 1 [file Image3.TIFF]

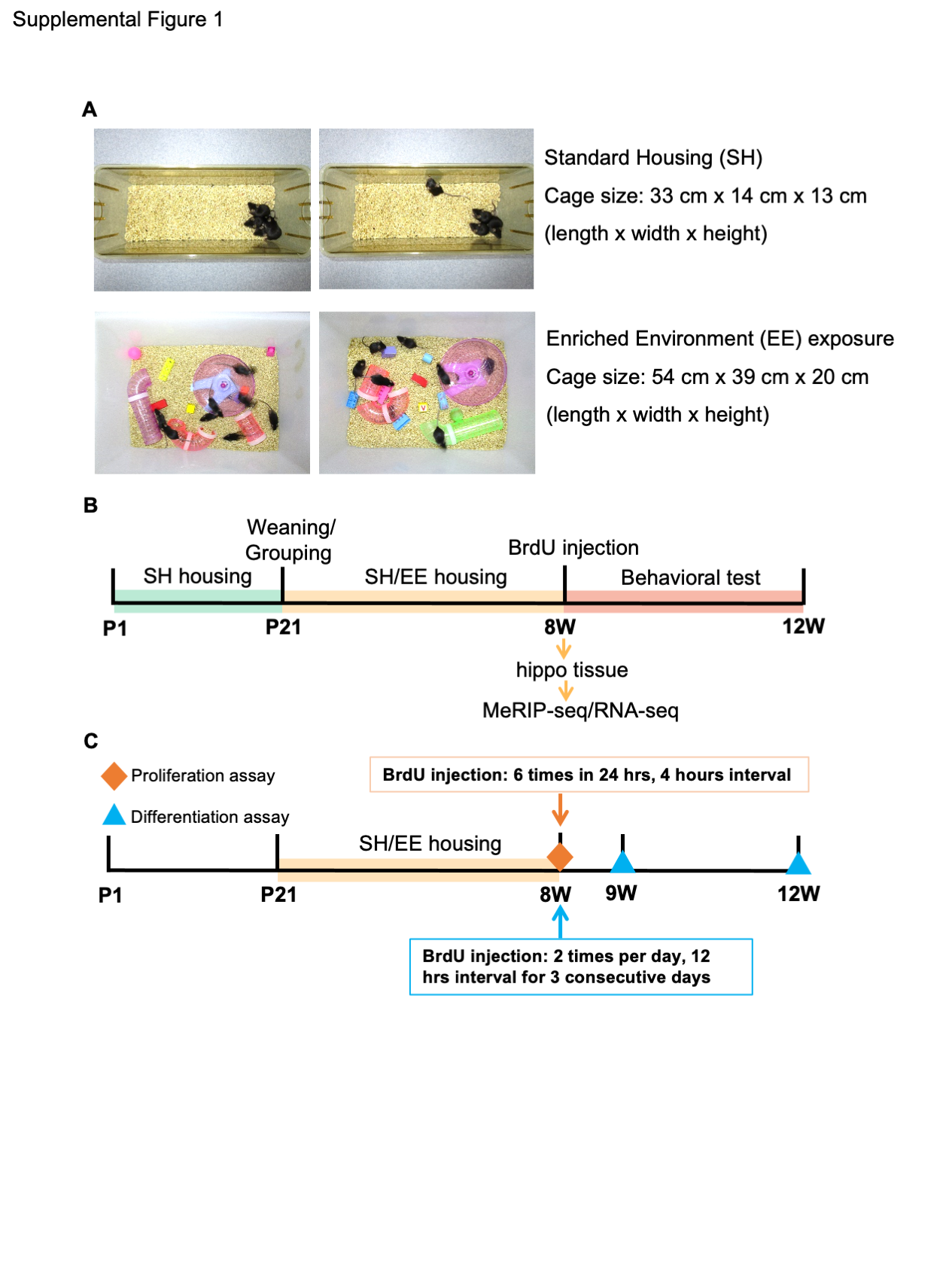

Supplement: Supplementary file 3 [file Image1.TIFF]

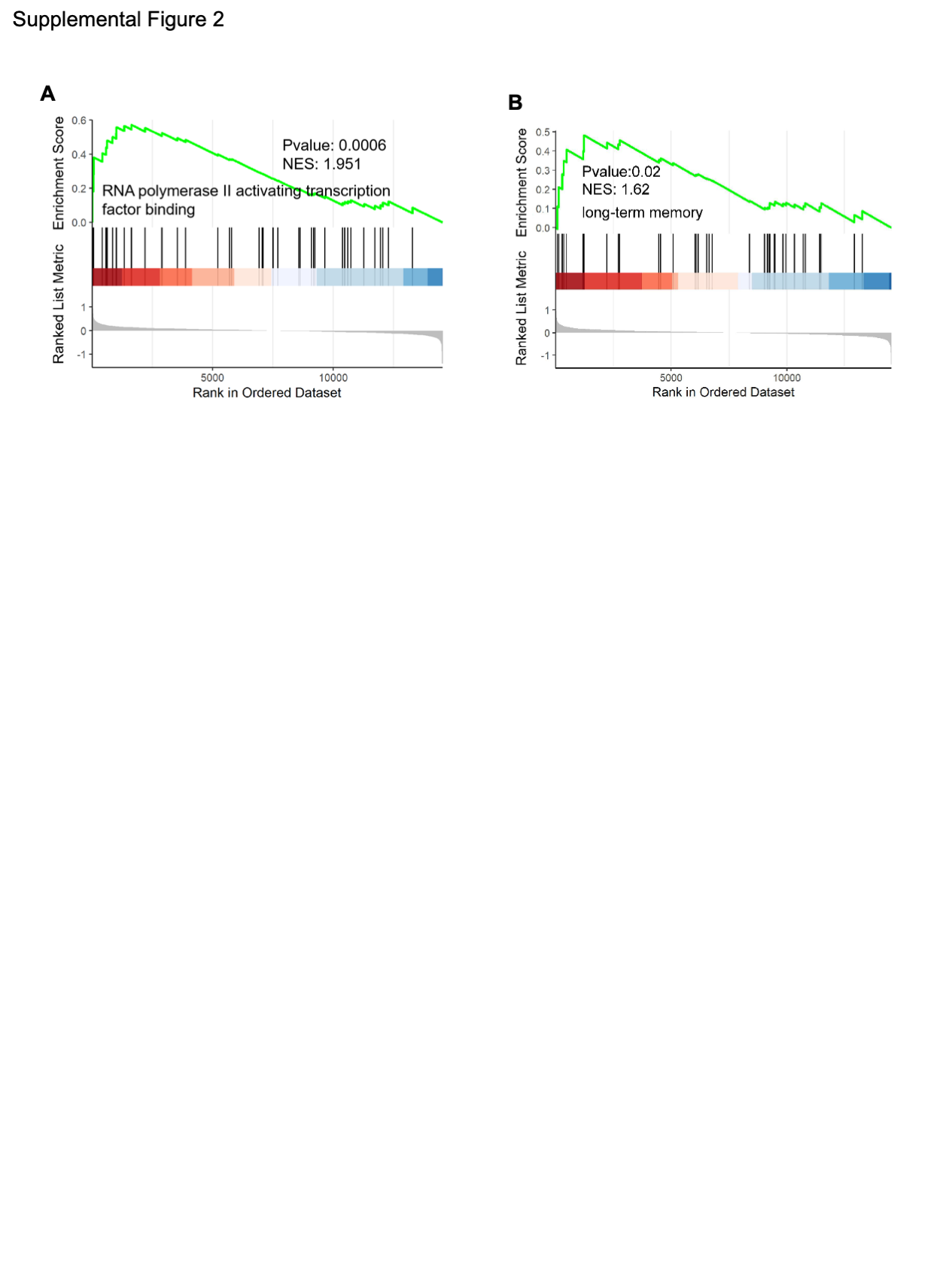

Supplement: Supplementary file 9 [file Image2.TIFF]

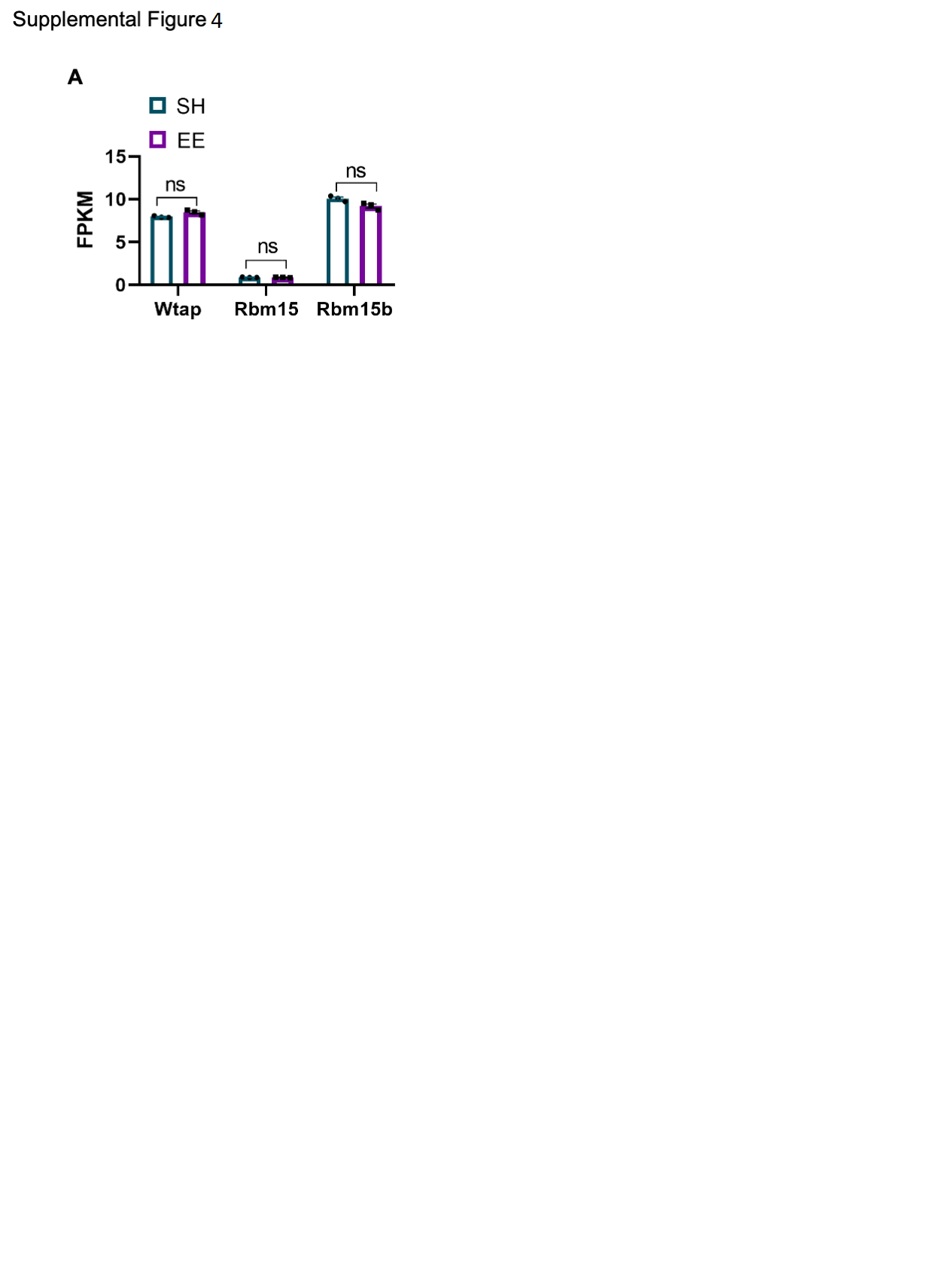

Supplement: Supplementary file 10 [file Image4.tiff]
